# A robust optimal control by grey wolf optimizer for underwater vehicle-manipulator system

**DOI:** 10.1371/journal.pone.0287405

**Published:** 2023-11-29

**Authors:** Yong Dai, Duo Wang, Fangyu Shen

**Affiliations:** 1 School of Automation and Electrical Engineering, Shenyang Ligong University, Shenyang, China; 2 Science and Technology Development Corporation, Shenyang Ligong University, Shenyang, China; 3 Shenyang Aircraft Company Limited, Aviation Industry Corporation of China, Ltd. (AVIC), Shenyang, China; University of Hull, UNITED KINGDOM

## Abstract

Underwater vehicle-manipulator system (UVMS) is a commonly used underwater operating equipment. Its control scheme has been the focus of control researchers, as it operates in the presence of lumped disturbances, including modelling uncertainties and water disturbances. To address the nonlinear control problem of the UVMS, we propose a robust optimal control approach optimized using grey wolf optimizer (GWO). In this scheme, the nonlinear dynamic model of UVMS is deduced to a linear state-space model in the case of the lumped disturbances. Then, the GWO algorithm is used to optimize the Riccati equation parameters of the *H*∞ controller in order to achieve the *H*∞ performance criterion, such as stability and disturbance rejection. The optimization is performed by evaluating the performance of the closed-loop UVMS in real-time comparison with the popular artificial intelligent algorithms, such as as ant colony algorithm (ACO), genetic algorithm (GA), and particle swarm optimization (PSO), using feedback control from the physical hardware-in-the-loop UVMS platform. This scheme can result in improved *H*∞ control system performance, and it is able to ensure that UVMS has strong robustness to these lumped disturbances. Last, the validity of the proposed scheme can be established, and its performance in overcoming modeling uncertainties and external disturbances can be observed and analyzed by performing the hardware-in-the-loop experiments.

## Introduction

Nowadays, underwater vehicle-manipulator systems (UVMS) have become a crucial and functional tool for humans to perform complex tasks in water [1]. In order to effectively control the UVMS, it is necessary to first build its dynamic model. This is because the dynamic model provides a mathematical representation of the system’s behavior, which can then be used to design effective control strategies and algorithms. However, as is widely recognized, creating an accurate dynamic model for UVMS remains a challenging task that requires precise determination and calculation of its parameters. Despite advancements in technology and methodology, developing a dynamic model that truly reflects the behavior of the UVMS continues to be a formidable undertaking, requiring a high degree of skill, expertise, and resources. This difficulty arises from the complexities inherent in the system and the dynamic environment in which it operates, making the process of obtaining accurate parameters a labor-intensive and time-consuming effort. Therefore, in some real-world applications of UVMS, approximations are made in the modeling process to simplify implementation. In addition to the difficulties in modeling, the presence of external disturbances in the underwater environment must also be acknowledged and taken into consideration. These disturbances, such as undercurrents, waves, and motion damping, are a ubiquitous aspect of the aquatic environment and can significantly impact the behavior and functionality of the UVMS. To address these modeling uncertainties and external disturbances, it is imperative that the UVMS control technique be designed with robustness and adaptability. This is crucial in order to maintain stability and ensure safe and effective operation in the face of these challenges. The need for robust and adaptive control techniques for the UVMS is of utmost urgency, and it is imperative that they be proposed and designed.

There have been a variety of control methods [2, 3] proposed for UVMS by scholars in the field, including proportional-integral-derivative (PID) [4, 5], expert system [6, 7], fuzzy control theory [8], active disturbance rejection control (ADRC) [9], model predictive control (MPC) [10–12], neural network control [13], sliding mode control (SMC) [14, 15], etc. However, many of these control theories suffer from limitations, such as ignoring disturbances, losing optimal control performance, reliance on accurate modeling, or high calculation complexity. This highlights the need for the continued development of more effective and robust control techniques for the UVMS that can handle the challenges of the underwater environment.

The *H*∞ controller is a type of optimal control approach that was quickly studied in the late 1970s to early 1980s [16]. It is specifically designed to handle disturbances and uncertainties in control systems, making it an effective solution for dealing with the complexities of many real-world systems. The *H*∞ controller uses mathematical min-max optimization techniques to find the optimal control inputs that minimize the impact of disturbances and uncertainties on the performance of the controlled system. By doing so, it can ensure that the control system operates as close as possible to its desired behavior, even in the presence of unpredictable or unmodeled disturbances and uncertainties. Currently, there are numerous research efforts focused on robust *H*∞ control of robots. For instances [17], proposed an *H*∞ control approach for a 6-degree-of-freedom (DOF) manipulator, and the proposed control law is effective for optimizing settling time, overshoot and steady state error for each joints [18], proposed a MATLAB-based structured *H*∞ control approach for the position control of a 4-DOF serial arm studied by Simulink [19], has proposed an *H*∞ control approach for the multi-DOF arm with flexible and stable joints [20], has proposed an asynchronous *H*∞ continuous control approach for the mode-dependent switched mobile robot system [21], has proposed an *H*∞ control of a flexible and stable cable-driven parallel robot [22], has proposed an *H*∞ feedback control approach for the underwater vehicle systems with various communication topology and external disturbances. However, it is noted that the *H*∞ controllers are typically designed and solved offline, meaning that the control design process occurs before the actual deployment of the system [23]. The design process involves finding an approximate solution of the Riccati equation, which is a mathematical equation that is central to the *H*∞ control method. The approximation solution is used to calculate the feedback gain matrix that represents the controller’s performance and robustness trade-off. Despite the widespread use of *H*∞ controllers, the solution of the Riccati equation can be computationally challenging and may not always converge for large-scale systems like the multi-input and multi-output UVMS. As a result, alternative methods for designing *H*∞ controllers for UVMS are being actively researched and developed. The prior research [24] is the only known discussion of *H*∞ control for UVMS, to the best of your knowledge. It has proposed a robust *H*∞ controller for UVMS using an extended Kalman filter (EKF). However, the calculation efficiency of this scheme has been found to be poor due to its reliance on the Kleinman [23] approximation solution of the Riccati equation. The Kleinman approximation is known to have limitations in terms of calculation efficiency, especially for large-scale systems like UVMS. As a result, the scheme proposed in [24] may not be suitable for real-time control of UVMS, particularly for systems that require fast convergence and efficient calculation. These limitations highlight the need for further research to develop more effective and efficient *H*∞ control methods for UVMS.

Recently, artificial intelligence algorithms have been introduced as alternatives to traditional control methods for various applications. Some of these algorithms include, ant colony algorithm (ACO), grey wolf optimizer (GWO), particle swarm optimization (PSO), and genetic algorithm (GA), etc [25]. These algorithms are based on bio-inspired optimization techniques, and they can be used to search for optimal control solutions by imitating the behavior of social animals or natural systems. They are becoming increasingly popular due to their ability to effectively handle complex and highly nonlinear control problems, and they have the potential to be used for developing more effective and efficient control solutions for the *H*∞ optimal control of UVMS. Among them, the GWO algorithm, first developed by Seyedali Mirjalili et al. in 2014, has become a well-known optimization method in the field. The mechanism of GWO involves simulating the natural behavior of grey wolves in their leadership hierarchy and hunting process [26, 27]. In particular, the algorithm employs four types of grey wolves, alpha, beta, delta, and omega, to simulate the strict social dominant hierarchy observed in wolf packs. Additionally, the three main steps of hunting, tracking and pursuing prey, encircling and harassing prey until it stops moving, and finally attacking, are implemented to perform optimization [26]. This unique approach has been shown to be both efficient and effective in solving a variety of optimization problems, making it a reliable and trustworthy optimization technique. The research in [28] firstly proposed the use of the GWO to optimize an *H*∞ controller for controlling UVMS in the presence of underwater disturbances. This method is based on solving linear matrix inequalities (LMI) by the GWO, which can be a complex operation. To address this challenge, we will propose transforming the LMI into a Riccati equation, providing a simpler and more intuitive solution. The proposed technique is considered a valuable motivation for future research. While the Riccati equation in *H*∞ controllers has been solved using various artificial intelligence algorithms [29, 30], this is the first time that the GWO has been used for this purpose.

Inspired from the above documents, the following contributions are given in this paper.

1. First, the proposed robust optimal *H*∞ controller in this paper utilizes the well-known GWO to optimize its parameters. The control scheme is specifically designed for the UVMS, which operates in the presence of lumped disturbances such as unmodeled uncertainties and external disturbances. The proposed control scheme takes advantage of the optimization capability of the GWO algorithm to determine the optimal parameters of the *H*∞ controller, which are used to achieve the desired performance criteria of stability and disturbance rejection.
2. Second, the optimization is based on a linear state-space model of the nonlinear UVMS, which enables the evaluation of the closed-loop system in real time. Furthermore, the proposed control scheme is compared with other popular artificial intelligence algorithms, such as PSO, ACO, and GA, using a hardware-in-the-loop UVMS platform, demonstrating its improved performance and robustness against lumped disturbances. The validity of the proposed scheme is established through hardware-in-the-loop experiments, which provide insight into its ability to overcome modeling uncertainties and external disturbances for UVMS.
3. Third, the aim of designing the robust optimal *H*∞ controller optimized by GWO for the UVMS is to address the nonlinear control problem of UVMS, which is commonly used in underwater operations and is subject to lumped disturbances such as unmodeled uncertainties and external disturbances. The proposed scheme improves the control system performance of UVMS and enhances its robustness to these disturbances, thereby enabling efficient and robust underwater operations.

This paper is organized below. Firstly, we will introduce the dynamic modeling of UVMS, including the linearized UVMS without and with lumped disturbances of the UVMS. Secondly, the design of the GWO-robust *H*∞ controller for UVMS is presented, and we will give its detailed theoretical statements. Thirdly, the simulation experiment is performed to verify the proposed control scheme’s perfomance for UVMS. Finally, conclusions are made.

## Linearized dynamic system analysis of UVMS

**Fig 1:**
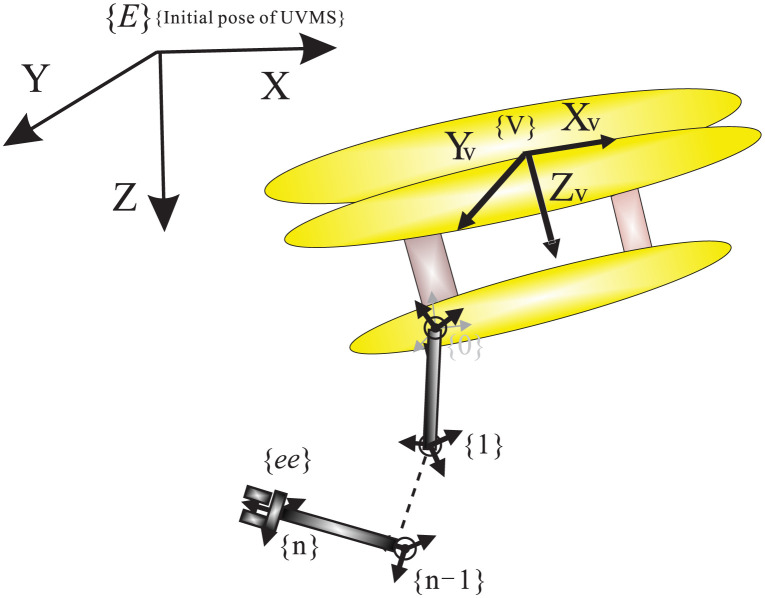
The basic structure of the UVMS.

The basic structure of the UVMS can be depicted in Fig 1. Assuming no external disturbances, the dynamic equation of the UVMS is given as follows [1]:

$$\begin{array}{c} M(q)a+C(q,v)v+D(q,v)v+G(q)=\tau \end{array}$$ (1)

In this equation, *q*, *v*, and *a* represent the position, velocity, and acceleration of the UVMS, respectively. The vector *q* = [*q*_*v*_, *q*_*m*_]^*T*^ is composed of the underwater vehicle state *q*_*v*_ = [*q*_1_, *q*_2_, ⋯, *q*_6_]^*T*^ and the underwater manipulator state *q*_*m*_ = [*q*_6+ 1_, *q*_6+2_, ⋯, *q*_6+*n*_]^*T*^. The inertia matrix, which incorporates the added mass terms, is represented by *M*(*q*). The Coriolis and centripetal terms are denoted by *C*(*q*, *v*). The hydrodynamic and motion damping matrix are represented by *D*(*q*, *v*). The effects of gravity and buoyancy are represented by *G*(*q*). The vector *τ* represents the forces, moments, and joint torques.

In an actual dynamic control system, it is challenging to accurately determine the dynamic model Eq (1), as the internal uncertainties and external disturbances are often unknown. These uncertainties and disturbances can be broadly categorized into two types [24]:

(a) Uncertainties in modeling parameters, such as mass, length, inertia, and centroid, among others.
(b) External water disturbances, including undercurrents, waves, and motion damping, among others.

By defining *C*(*q*, *v*)*v*+ *D*(*q*, *v*)*v*+ *G*(*q*) = *H*(*q*, *v*) and unifying all internal and external disturbances into a single bounded unknown lumped disturbance *τ*_*d*_, where $\tau_{d}\in W$ and $W=\{\tau_{d},∥\tau_{d}∥\leq \omega_{max}\}$ with a maximum bounded assumption *ω*_*max*_, the dynamic equation of the UVMS can be expressed as:

$$\begin{array}{c} M\left(q\right)\dot{v}+H\left(q,v\right)=\tau +\tau_{d} \end{array}$$ (2)

The system can be given by a state-space form as follows:

$$\begin{array}{c} \left\{ \begin{array}{c} \dot{q}=v\\ \dot{v}=M{\left(q\right)}^{-1}\left(-H\left(q,v\right)+\tau +\tau_{d}\right) \end{array}\right. \end{array}$$ (3)

Next, the proposed control law is applied:

$$\begin{array}{c} \tau =M\left(q\right)\left(C_{1}v+C_{2}q\right)+H\left(q,v\right)+M\left(q\right)u, \end{array}$$ (4)

where *C*_1_ and *C*_2_ are matrices representing velocity and position error gains, respectively. Substituting this control law into Eq (3) yields:

$$\begin{array}{c} \left\{ \begin{array}{c} \dot{q}=v\\ \dot{v}=C_{1}v+C_{2}q+u+M{\left(q\right)}^{-1}\tau_{d} \end{array}\right. \end{array}$$ (5)

By defining $x=[\begin{array}{c} q\\ v \end{array}]$, $A=[\begin{array}{cc} 0 & I\\ C_{2} & C_{1} \end{array}]$, $L=[\begin{array}{c} 0\\ I \end{array}]$, $B=[\begin{array}{c} 0\\ I \end{array}]$, Eq (5) can be expressed in state-space model form as follows:

$$\begin{array}{c} \dot{x}=Ax+Bu+LM{\left(q\right)}^{-1}\tau_{d} \end{array}$$ (6)

Letting $\tilde{d}=M{\left(q\right)}^{-1}\tau_{d}$ represent the lumped disturbances (including the internal uncertainties and external disturbances), we obtain the following linear state-space model under the condition of disturbances:

$$\begin{array}{c} \dot{x}=Ax+Bu+L\tilde{d} \end{array}$$ (7)

Thus, the nonlinear dynamic equations of UVMS have been successfully reduced to a linear state-space model, making it easier to design a *u* control law from an *H*∞ controller.

## The design of the GWO-robust *H*∞ controller for UVMS

The renowned GWO algorithm [26] is employed to address this optimization problem. It has been demonstrated to be an effective optimization technique similar to algorithms such as PSO, ACO, and GA. The GWO is a meta-heuristic algorithm that is inspired by the hunting behavior of wolf packs. The wolves in the pack are classified into four classes: *α* (the leader), *β* and *δ* (the followers), and *ω* (the rest). In the context of engineering problems, the optimal solution is considered to be the leader (corresponding to class *α*), sub-optimal solutions correspond to *β* and *δ*, and alternative solutions belong to *ω* [27].

The hunting process of wolves is comprised of three steps:

1. Tracking, pursuing, and approaching the target.
2. Circling and harassing the target until it stops moving.
3. Launching an attack.

The hunting actions of wolves can be described as follows:

$$\begin{array}{c} \begin{array}{cc} & \vec{D}=|\vec{C}\cdot \vec{X_{p}\left(t\right)}-\vec{X(t)}|\\ & \vec{X}\left(t+1\right)=\vec{X_{p}}\left(t\right)-\vec{A}\cdot \left(\vec{D}\right) \end{array} \end{array}$$ (8)

where *t* is the number of iterations, $\vec{a}$, $\vec{r_{1}}$ and $\vec{r_{2}}$ are coefficient vectors, $\vec{X_{p}}$ and $\vec{X}$ represent the prey position vector and the wolf position vector, respectively. The $\vec{a}$, $\vec{r_{1}}$ and $\vec{r_{2}}$ can be used to calculate the following parameters $\vec{A}$ and $\vec{C}$:

$$\begin{array}{c} \begin{array}{cc} & \vec{A}=2\vec{a}\cdot \vec{r_{1}}-\vec{a},\\ & \vec{C}=2\cdot \vec{r_{2}}, \end{array} \end{array}$$ (9)

During the iteration process, each exponent in $\vec{a}$ can be randomly chosen from the range 2 to 0, while the values of $\vec{r_{1}}$ and $\vec{r_{2}}$ are random vectors within the interval [0, 1].

In the GWO algorithm, the wolves in class *ω* follow the wolves in classes *α*, *β*, and *δ* to hunt and approach the target, as these classes are considered to be more capable of catching prey. Thus, the first three optimal solutions are saved in the project, while the other wolves update the current state based on the current optimal solution. This can be expressed by the following Eq (10):

$$\begin{array}{c} \begin{array}{cc} \vec{D_{\alpha }} & =|\vec{C_{1}}\cdot \vec{X_{\alpha }}-\vec{X}|\\ \vec{D_{\beta }} & =|\vec{C_{2}}\cdot \vec{X_{\beta }}-\vec{X}|\\ \vec{D_{\delta }} & =|\vec{C_{3}}\cdot \vec{X_{\delta }}-\vec{X}|\\ \vec{X_{1}} & =\vec{X_{\alpha }}-\vec{A_{1}}\cdot (\vec{D_{\alpha }})\\ \vec{X_{2}} & =\vec{X_{\beta }}-\vec{A_{2}}\cdot (\vec{D_{\beta }})\\ \vec{X_{3}} & =\vec{X_{\delta }}-\vec{A_{3}}\cdot (\vec{D_{\delta }})\\ \vec{X}\left(t+1\right) & =\frac{\vec{X_{1}}+\vec{X_{2}}+\vec{X_{3}}}{3} \end{array} \end{array}$$ (10)

**Fig 2:**
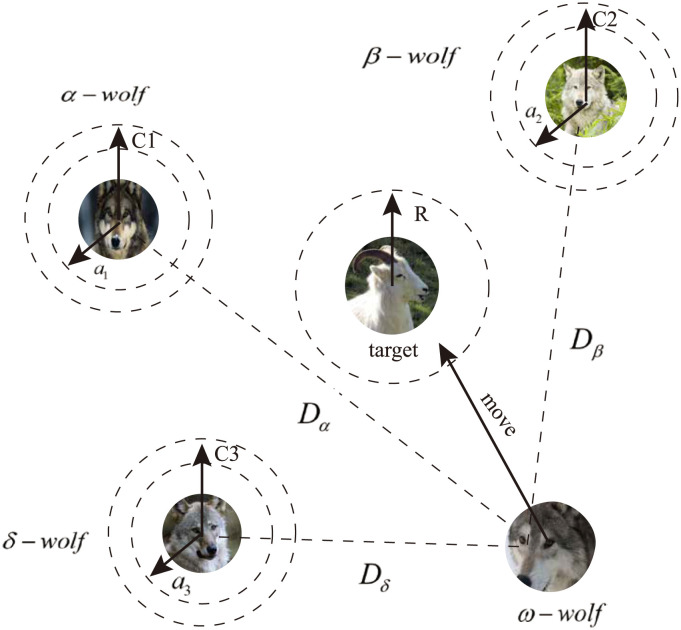
The position updating strategy in GWO.

Through Eq (10), the wolves update their positions in the n-dimensional space according to *α*, *β*, and *δ*, as shown in Fig 2. The final position remains within the range defined by *α*, *β*, and *δ*, while the other wolves in *ω* continue to update their positions randomly around the target according to *α*, *β*, and *δ*, and they continuously estimate the target position.

The process of the GWO algorithm is described below, with a flowchart of the process shown in Fig 2:

1. Generate a random wolf pack (candidate solutions).
2. Update the position of each wolf in *ω* according to the target position estimated by *α*, *β*, and *δ*.
3. Decrease the value of $\vec{a}$ linearly from 2 to 0 in the iterative process, which emphasizes the hunting or attack. When $\vec{a}$ is greater than 1, the wolf moves away from the target, and when $\vec{a}$ is less than 1, it gets closer to the target, thereby avoiding local stagnation.
4. Terminate the GWO algorithm when the stopping criteria are met.

The robust *H*∞ control theory was proposed by Canadian scientists Zames et al. [31]. The standard control cost function for this theory is given by:

$$\begin{array}{l} J(t)=\frac{1}{2}\int_{0}^{T}\left[y^{T}(t)y(t)+\rho^{2}{\tilde{d}}^{T}(t)\tilde{d}(t)-ru^{T}(t)u(t)\right]dt\\ r,\rho >0,y(t)=Cx(t) \end{array}$$ (11)

The significance of the standard control cost function lies in the fact that it represents a zero-sum game problem, where the disturbance variable $\tilde{d}\left(t\right)$ tries to maximize the objective function *J*(*t*) while the control signal *u*(*t*) tries to minimize it. To solve this problem, a Riccati equation can be used to obtain an approximation. The general method for solving the Riccati equation is the Kleinman method [23]. However, in this paper, we introduce a novel GWO method for solving the Riccati equation in the robust *H*∞ control of UVMS.

The conventional Riccati equation arrived from the robust *H*∞ control is generally stated as [24]:

$$\begin{array}{c} A^{T}P+PA=-Q+P(\frac{2}{r}BB^{T}-\frac{1}{\rho^{2}}LL^{T})P \end{array}$$ (12)

where given the positive definite matrix *Q* and coefficients *r* and *ρ* to solve a positive definite matrix *P*. The robust *H*∞ controller used to control the UVMS system is obtained by

$$\begin{array}{c} u=-\frac{1}{r}B^{T}Px. \end{array}$$ (13)

**Fig 3:**
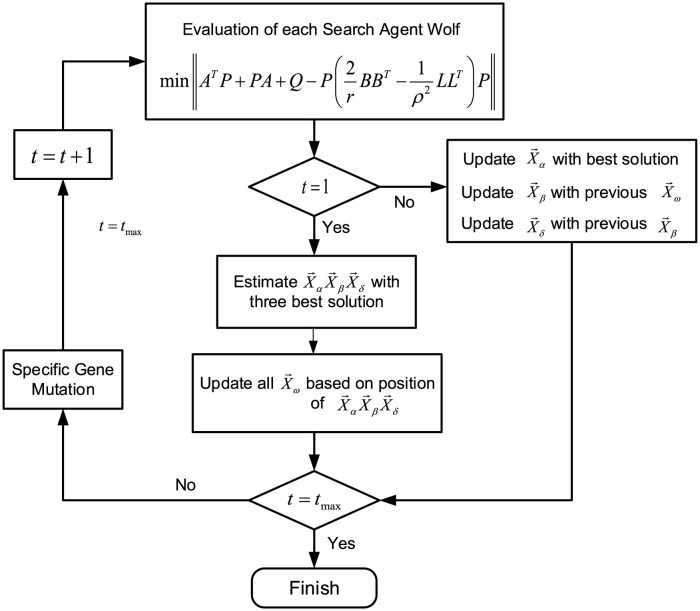
The flowchart of the GWO algorithm.

**Fig 4:**
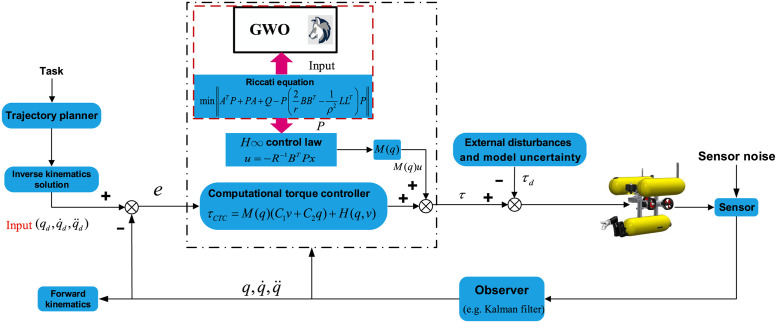
The proposed robust *H*∞ controller based on GWO for UVMS.

The utilization of the proposed method is depicted in Fig 3 for better illustration. The objective of incorporating the GWO algorithm to optimize the conventional Riccati equation is to determine the optimal solution for the positive definite matrix *P*. The method of optimization is outlined in Algorithm 1, and a comprehensive visualization of the control system design for the UVMS is presented in Fig 4. The control action comprises two parts: the GWO-*H*∞ controller and the computational torque controller (CTC) *τ*_*CTC*_ based on the nominal dynamic system.

**Algorithm1**

**Step1** Re-arrange the conventional Riccati equation into a min-function type by:

minimizing $∥A^{T}P+PA+Q-P(\frac{2}{r}BB^{T}-\frac{1}{\rho^{2}}LL^{T})P∥$. The variable to be optimized using GWO is a positive definite matrix *P*. For ease of implementation, set *Q* to be the identity matrix, *r* = 1, and *ρ* = 1.

**Step2** The matrix optimization is constructed as *P* = diag([*P*_1_, *P*_2_, …, *P*_*n*_]).

**Step3** Initialize the wolves’ positions randomly and give them proper quantities, and let a group of wolves represent a matrix *P* = diag([*P*_1_, *P*_2_, …, *P*_*n*_]).

**Step4** Select the optimal group of wolves as a proper solution of the minimized function in **Step1**.

**Step5** Use the optimal group of wolves as the center of the entire pack of wolves and update the positions of other wolves by moving them closer to the center in one step.

**Step6** Update the position of the new lead wolf according to the “winner takes all” rule and update the entire wolf system according to the “strong survive” mechanism.

**Step7** Check if the optimization goal of the min-function in **Step1** has been reached. If it has, then output the lead wolf’s position as the optimal solution of the problem. If not, return to **Step5**.

## Hardware-in-the-loop experiments

**Fig 5:**
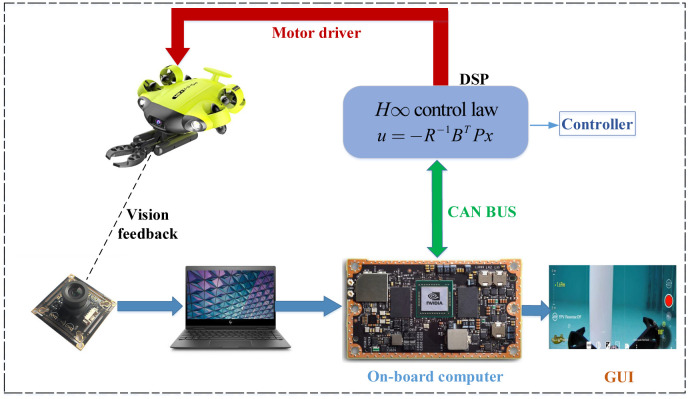
The hardware-in-the-loop experimental setup.

The hardware-in-the-loop experiment for the control of the UVMS (QYSEA FIFISH V6, with physical parameters listed in Table 1) is conducted using advanced simulation software, Simurv4.0 [1]. The whole control structure is given in Fig 5. The computer configuration used in the experiment features an AMD Ryzen R7-5800H CPU, a 512GB SSD hard disk with an upgradeable capacity of 1TB SSD, memory options of 32GB, and a standalone RTX3060 graphics card. The control of the UVMS is carried out through the Simurv4.0 simulation environment to the on-board computer, the Jetson TX2 embedded system, with the following parameters: CPU—a dual-core NVIDIA Denver 2 64-bit CPU combined with a quad-core Arm Cortex-A57 MPCore processor, Memory—4 GB 128-bit LPDDR4 51.2 GB/s, Storage—16 GB flash memory with an M.2 M KEY NVME solid state interface. The communication is CANBUS, and digital signal processing (DSP) of the type TMS320C5X is used for sending and analyzing the driver data. The vision feedback detector used in the system is a 4K mono-camera. The sample time chosen is 100 ms.

**Table 1:** Physical parameters of the UVMS.

parameters	dry weight[kg]	buoyancy[kg]
Link1	80.0	106.8
Link2	80.0	31.4
Link3	30.0	14.1
Link4	50.0	25.1
Link5	20.0	14.1
Link6	25.0	9.4
vehicle	223.9327	205.123

**Fig 6:**
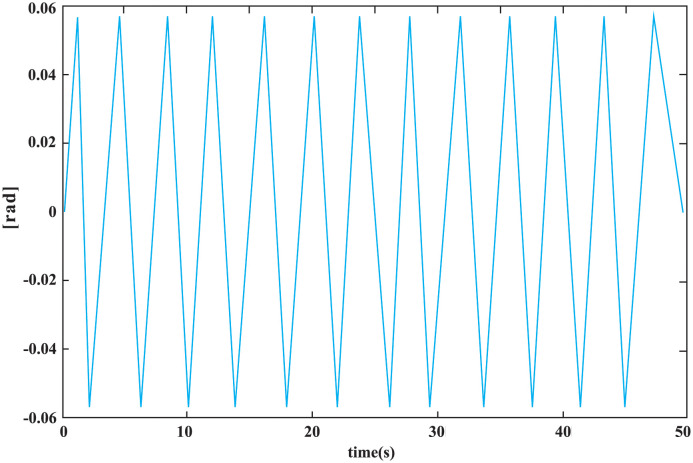
The system lumped disturbances assumed for the bounded unknown step signal of *H*∞ controller for each DOF.

A composite control scheme, combining a disturbance observer (DOB), an *H*∞ controller, and a GWO online solver, is applied to enhance the control performance of the UVMS in the face of dynamic uncertainties and unknown disturbances. The UVMS is initialized at a position of (0, 0, 0) and a manipulator configuration of $q_{m}=[\begin{array}{cccccc} 0 & -45 & -45 & 0 & 0 & 0 \end{array}]/180*\pi$, while its target position for the manipulator is set at (-0.6, 1.2, 4). The optimal matrix *P* = diag([*P*_1_, *P*_2_, ⋯, *P*_*n*_]) for the *H*∞ controller of the UVMS is found using GWO’s Algorithm 1, with randomly initialized artificial wolves and GWO parameters specified in Table 2. The inverse kinematics (IK) of the control goal task is obtained and fed into the Riccati equation of the *H*∞ controller, with GWO serving as the solver to obtain the online control actions for the UVMS. The robustness and adaptiveness of the UVMS are verified through the lumped disturbances depicted in Fig 5. The proposed control scheme is evaluated through realistic simulations and relevant experiments to assess its effectiveness and ability to provide optimal control action. Additionally, to assess the robustness and adaptiveness of the *H*∞ control, a bounded signal assumption of lumped disturbances is provided in Fig 6 for verification purposes.

**Table 2:** Initialization parameters of the GWO algorithm.

wolves quantity	*n* = 500
maximum iterations	*t*_max_ = 100
searching parameters $\vec{a}$	$\vec{a}=2.0\cdot I$
searching parameters $\vec{r_{1}}$	$\vec{r_{1}}=0.5\cdot I$
searching parameters $\vec{r_{2}}$	$\vec{r_{2}}=0.3\cdot I$

**Fig 7:**
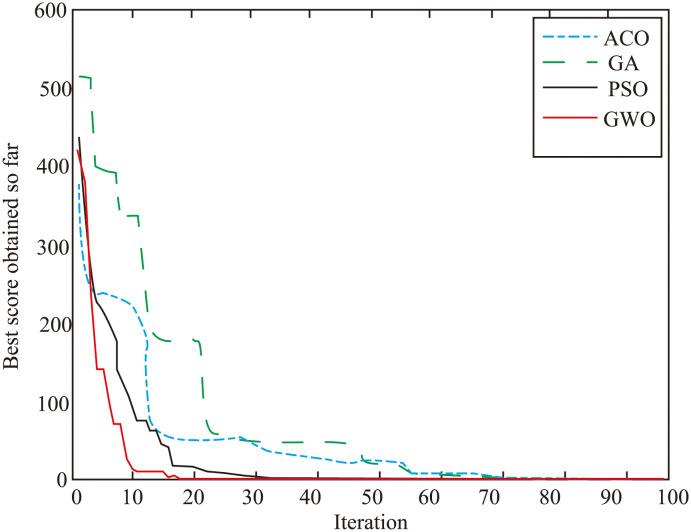
The optimization trend for each algorithm.

In order to determine the efficiency of the GWO algorithm in optimizing the performance of the *H*∞ controller, we compared it with other popular artificial intelligence algorithms such as PSO, ACO, and GA. We recorded the results of 200 trials to compare the efficiency of the GWO algorithm with other popular artificial intelligence algorithms such as PSO, ACO, and GA, and the data from all the trials is summarized in Table 3. The comparison was made to assess the performance of these algorithms in optimizing the Riccati equation of the *H*∞ controller. The best result among all the trials is presented in Fig 7, which has 100 iterations. The results of the comparison reveal that the GWO algorithm displays the fastest convergence rate in optimizing the Riccati equation, making it the best choice among the algorithms tested. The other algorithms, such as PSO, ACO, and GA, although popular, were found to not perform as well in this particular optimization task. After utilizing the GWO algorithm to optimize the *H*∞ controller, the best tuning results were achieved. The corresponding minimum value of the min-function, as shown in the equation below, was calculated to be 1.314.

$$\begin{array}{c} \begin{array}{cc} & \text{min}∥A^{T}P+PA+Q-P(2BB^{T}-LL^{T})P∥\\ & =1.314 \end{array} \end{array}$$ (14)

**Table 3:** Experimental comparison of different algorithms’ solutions to the Riccati equation.

Optimization	Algorithm	Mean	Variance	Maximum	Minimum
Eq (12)	GWO	0.24E + 04	1.15E + 03	1.48E + 04	1.10E + 03
	PSO	10.22E + 04	1.33E + 03	15.40E + 04	10.17E + 04
	GA	10.45E + 04	1.46E + 03	17.76E + 04	10.22E + 04
	ACO	10.28E + 04	1.11E + 03	16.30E + 04	10.10E + 03

The optimized value of the matrix *P* was determined to be diag([1.32 1.01 1.01 0.91 1.36 1.67 0.87 4.62 9.01 0.61 0.71 1.26]). With these parameters, the robust *H*∞ controller was then given by the equation $u=-\frac{1}{r}B^{T}Px$.

**Fig 8:**
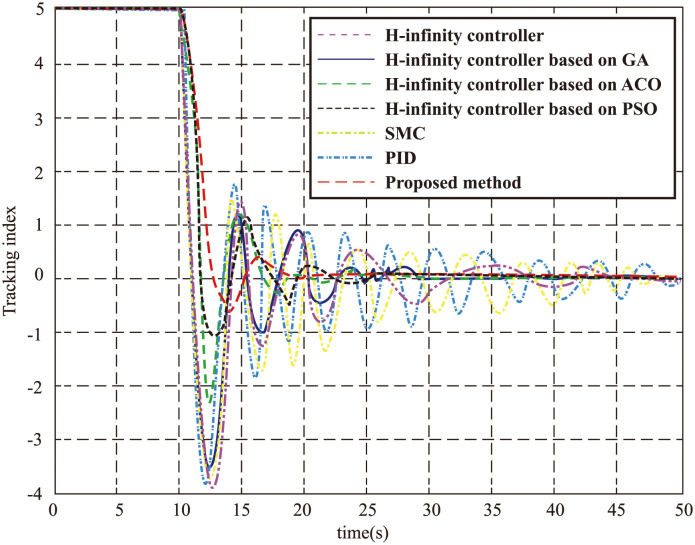
The tracking performance for each algorithm.

To further assess the performance of our proposed method in controlling the UVMS, we compare it with the control results obtained from other well-established techniques, such as the *H*∞ controller [24], the *H*∞ controller based on GA, the *H*∞ controller based on ACO, the traditional *H*∞ controller based on PSO, SMC and PID. The comparison results demonstrate that our proposed method surpasses other algorithms in terms of rapid response, tracking accuracy, system stability, and interference suppression. Furthermore, our method exhibits a more pronounced improvement over traditional non-optimized algorithms. This is attributed to the inherent issue of chaterring in SMC, which, despite its ability to overcome certain bounded interference, yields poor results as it lacks optimization capabilities. The classic PID method struggles to identify suitable parameters and fails to achieve superior control performance in the presence of real-time water flow changes. This is evident from the convergence output depicted in Fig 8, where our proposed method effectively tracks the target and converges to the reference, showcasing a significant improvement over other algorithms. This substantiates its efficacy in fulfilling the design requirements of the UVMS.

**Fig 9:**
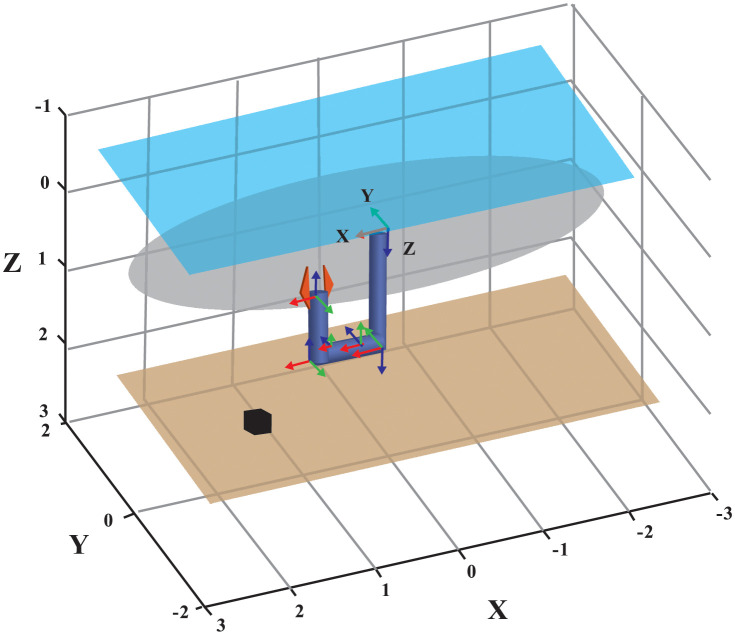
The initial position for the hardware-in-loop simulated UVMS.

**Fig 10:**
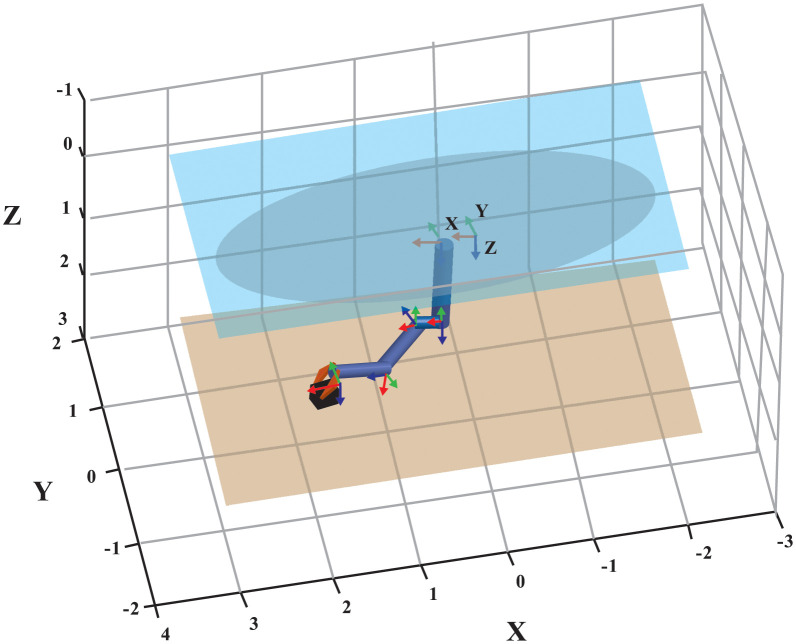
The target position for the hardware-in-loop simulated UVMS.

**Fig 11:**
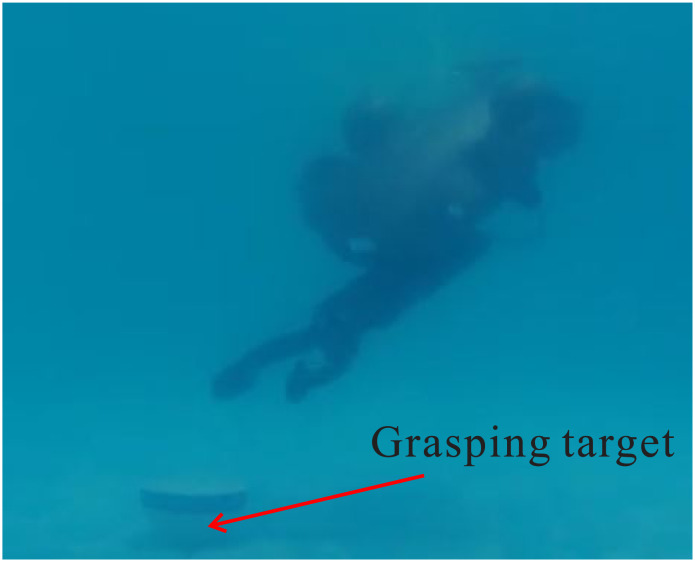
The tracking process of the experimental UVMS.

**Fig 12:**
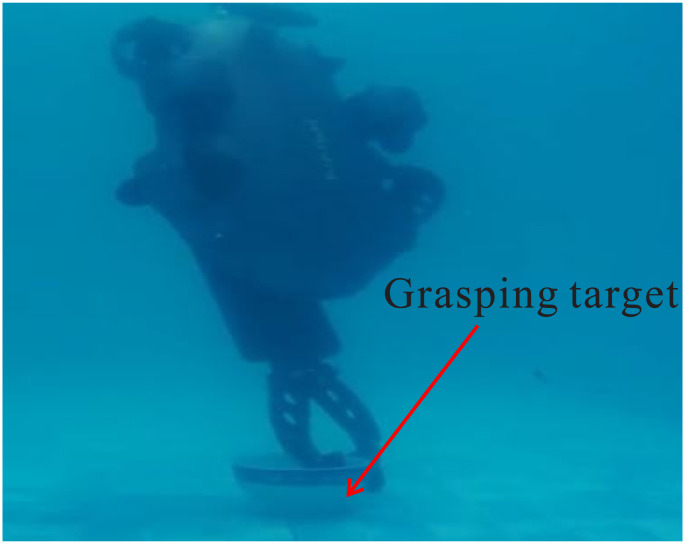
The final grasping of the experimental UVMS.

**Fig 13:**
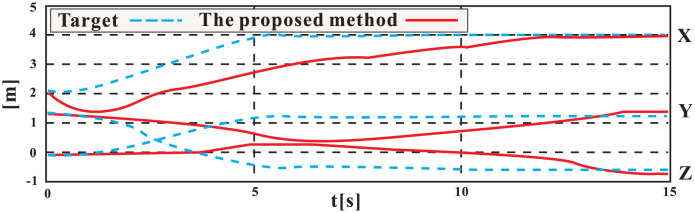
The position coordinates of the moving end-effector.

**Fig 14:**
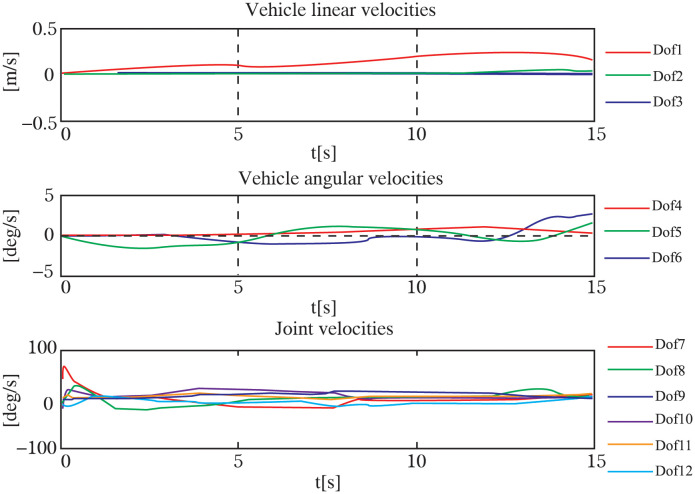
The velocity coordinates of each DOF for the motion trajectory.

**Fig 15:**
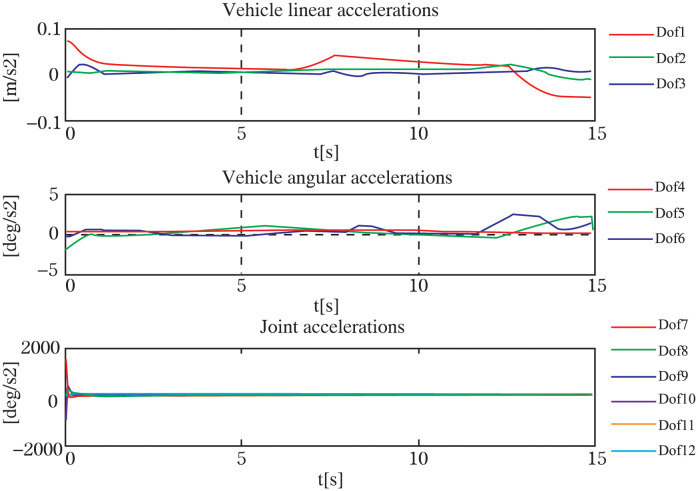
The acceleration coordinates of each DOF for the motion trajectory.

**Fig 16:**
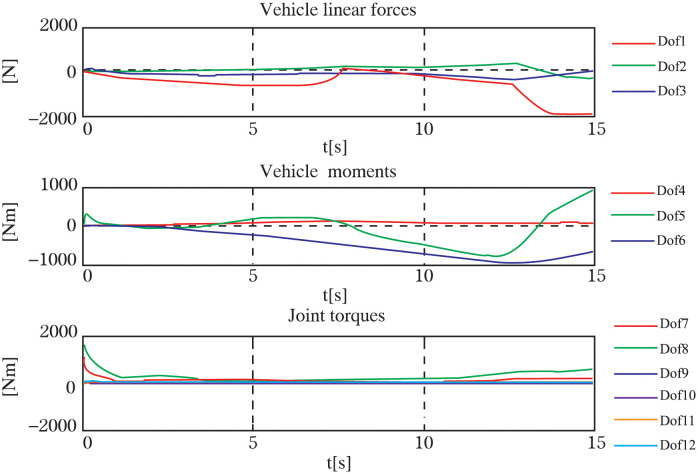
The force coordinates of each DOF for tracking control.

Throughout the experiment, the data was carefully recorded and analyzed to assess the performance of the control system. Utilizing the robust *H*∞ controller optimized by the GWO algorithm, the UVMS was able to successfully track and grasp the target. The entire control process can be observed in Figs 9–12, which demonstrate the UVMS’s ability to effectively maneuver and accurately track the target. Additionally, the detailed motion trajectory of the UVMS, including its position tracking, velocity variations, acceleration variations, and generalized forces, are shown in Figs 12–16. These figures clearly illustrate the effectiveness of the proposed control method, demonstrating its ability to accurately track the target and accomplish the task at hand. It can be concluded that the use of the GWO algorithm to optimize the *H*∞ controller results in a highly effective and efficient control system for the UVMS.

## Conclusion

In conclusion, the hardware-in-the-loop experiment of the GWO optimized *H*∞ controller for UVMS demonstrates the effectiveness of our approach. By transforming the Lagrange dynamic equation into a state-space linear model equation, the nonlinear UVMS control problem with lumped disturbances was effectively addressed. The GWO algorithm was chosen for its fast convergence trend in optimizing the traditional robust *H*∞ controller, which was successfully utilized to the control of the experimental nonlinear UVMS. The simulation results showed that the convergence speed of the control system met the engineering application standard, and the velocity, acceleration, and generalized forces from the degrees of freedom of the UVMS were all stable, smooth, and with minimal overshoot. This intelligent controller provides a promising solution for controlling nonlinear UVMS in the future. Further tests will be conducted to fully assess its performance.
